# Accuracy of Multiple Pour Cast from Various Elastomer Impression Methods

**DOI:** 10.1155/2016/7414737

**Published:** 2016-12-14

**Authors:** Satheesh B. Haralur, Majed Saad Toman, Abdullah Ali Al-Shahrani, Abdullah Ali Al-Qarni

**Affiliations:** ^1^Department of Prosthodontics, College Of Dentistry, King Khalid University, Abha, Saudi Arabia; ^2^College Of Dentistry, King Khalid University, Abha, Saudi Arabia

## Abstract

The accurate duplicate cast obtained from a single impression reduces the profession clinical time, patient inconvenience, and extra material cost. The stainless steel working cast model assembly consisting of two abutments and one pontic area was fabricated. Two sets of six each custom aluminum trays were fabricated, with five mm spacer and two mm spacer. The impression methods evaluated during the study were additional silicone putty reline (two steps), heavy-light body (one step), monophase (one step), and polyether (one step). Type IV gypsum casts were poured at the interval of one hour, 12 hours, 24 hours, and 48 hours. The resultant cast was measured with traveling microscope for the comparative dimensional accuracy. The data obtained were subjected to Analysis of Variance test at significance level <0.05. The die obtained from two-step putty reline impression techniques had the percentage of variation for the height −0.36 to −0.97%, while diameter was increased by 0.40–0.90%. The values for one-step heavy-light body impression dies, additional silicone monophase impressions, and polyether were −0.73 to −1.21%, −1.34%, and −1.46% for the height and 0.50–0.80%, 1.20%, and −1.30% for the width, respectively.

## 1. Introduction

Indirect restorations constitute the large part of the restorative and prosthetic treatment plan [1]. The accurate and dimensionally stable impressions are indispensable for the fabrication of indirect restoration. An impression is a record, a facsimile of the oral tissues recorded at an unstrained or in the various positions of displacement [2]. The excellent tooth preparation is of little value unless its details are accurately transferred to the dental laboratory. The precise working cast facilitates fabrication of the ideal restoration for the technician. The previous studies observed the inaccurate and poorly adapted restoration that led to compromised esthetics along with the biological failure of fixed partial denture [3].

Over the years, the dental materials are developed to improve their dimensional stability, reproducibility, and handling. The impression methods are also evolved to complement the recent material advancement to improve the impression accuracy. The selection of the impression methods is entirely at the discretion of the dentist. It is mainly influenced by the clinical conditions and variations. The elastomeric impression materials are most widely used in the restorative dentistry due to multiple advantages like good reproducibility, elastic recovery, and dimensional stability [4]. The majority of impression techniques are comprised of different viscosity impression materials. The high viscosity material is used as a preliminary impression and low viscosity material is utilized to record the finer details of preparation. Both single-stage and two-stage impressions are known to provide accurate impressions [5–7].

The accuracy of an impression with repeated pour is of great advantage for the clinician and laboratory technicians. It reduces the professional clinical time, patient inconvenience, and extra material cost. The change of interabutment distance during cast sectioning and loss of gingival reference during die ditching necessitate the additional intact duplicate cast for verification of finished restoration.

The dimensional accuracy of the cast from repeated pour is influenced by the extent of elastic recoil from distortion during cast retrieval and continued polymerization shrinkage [8]. These factors are greatly affected by the thickness/nature of impression material and stress induced during impression procedures [9]. Hence, it is important to understand the role of different impression methods on the dimensional accuracy of the resultant dies, especially on the multiple pours. The objective of the study was to compare the dimensional accuracy of the cast obtained from different impression methods on the multiple pours of a single impression at various time intervals. The impression methods evaluated in the study were two-stage putty relined with light body additional polyvinyl siloxane, single-stage heavy-light body additional silicone, single-stage monophase, and single-step polyether.

## 2. Materials and Methods

**Figure 1:**
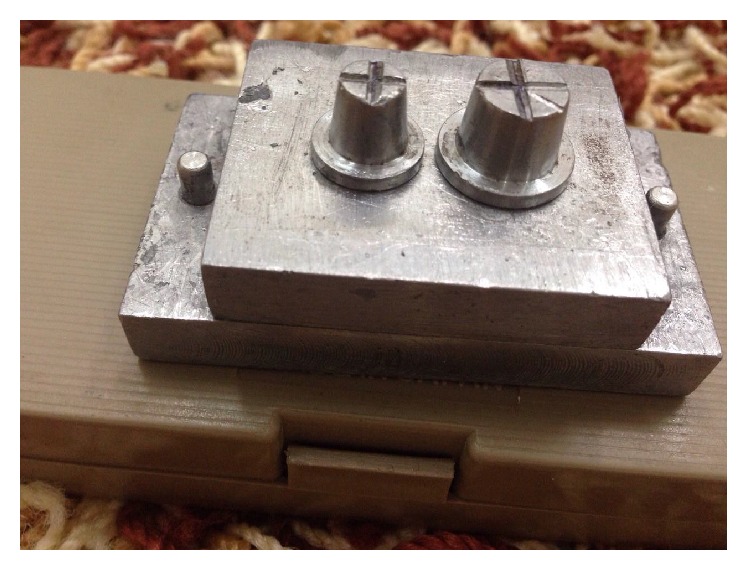
Stainless steel master cast used in the study.

**Figure 2:**
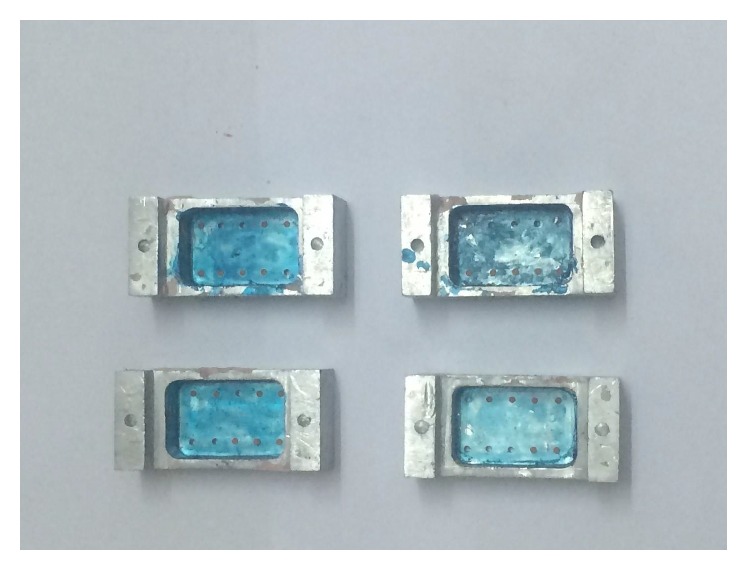
Aluminum impression trays used in the study.

The institutional ethical committee approval was obtained for the study research proposal (SRC/REG/2014-2015/17). The stainless steel working cast model assembly consisting of two abutments and one pontic area was fabricated (Figure 1). The stainless steel abutments were made to simulate the full veneer crown preparation with uniform 2 mm shoulder finish line and 6-degree taper. The first abutment was 8.2 mm in height and 9.9 mm in diameter at the occlusal surface. The measurement at the same location for the second abutment was 7.6 mm and 7.04 mm, respectively. The interabutment distance was 14.93 mm. The occlusal surface of the abutment was flat with reference cross groove for precise measurement. Two sets of perforated, rectangular custom aluminum trays were fabricated for making an impression (Figure 2).

The first set of six impression trays were used for putty wash impression technique; they possessed uniform spacer width of five mm. Another set of impression trays were having two mm uniform spacer area for a heavy body-light body one-step impression method. The impression tray stopper on working cast model base maintained the uniform space for the impression. The corresponding pin on the cast and groove in the impression trays was helpful in accurate position and immobilization of trays during impression procedure.

The impression methods compared in the study were the two-step putty reline technique, one-step heavy-light body, and one-step monophase polyvinyl siloxane material. The study also included the one-step monophase polyether impression.

The impression procedures were conducted at controlled room temperature (25 ± 10°C) and handled according to the manufacturer's instruction. The silicone/polyether tray adhesive applied over the tray allowed it to dry for 10 minutes prior to the impression procedure. Weighing the materials in precision scale established the accurate base and catalyst proportion; twenty gm of each constituent was used during the study. Two mm uniform spacer thickness was obtained by adapting vacuum resin sheet over model for two-stage putty wash technique. At the first stage, the mixed putty was loaded into the tray and allowed it to set over the cast for four minutes. The second stage involved the removal of the spacer and relining the preliminary putty impression base with light body elastomers.

**Figure 3:**
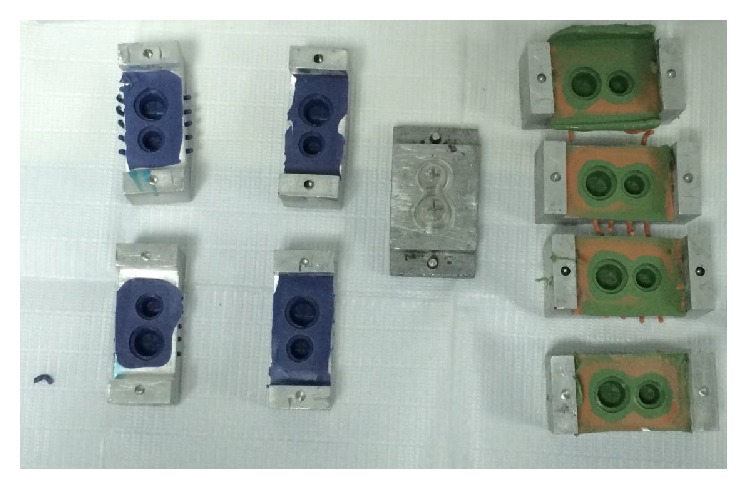
Polyether and putty reline impression made in custom trays.

The single-stage heavy-light body impression was accomplished by simultaneous loading of the heavy consistency material to the tray, and light viscosity material was spread over the stainless steel die. The impression tray was placed over the cast; the layer of the impression materials was allowed to set for 5 minutes. The single-stage impression from polyvinyl siloxane monophase and polyether single consistency impression were done in a similar procedure except for the overlying light body impression material (Figure 3).

After impression procedure, all polyvinyl siloxane impressions were rinsed in water and disinfected with sodium hypochlorite (1 : 10) immersion for 3 minutes. The polyether impressions were disinfected with sodium hypochlorite spray.

**Figure 4:**
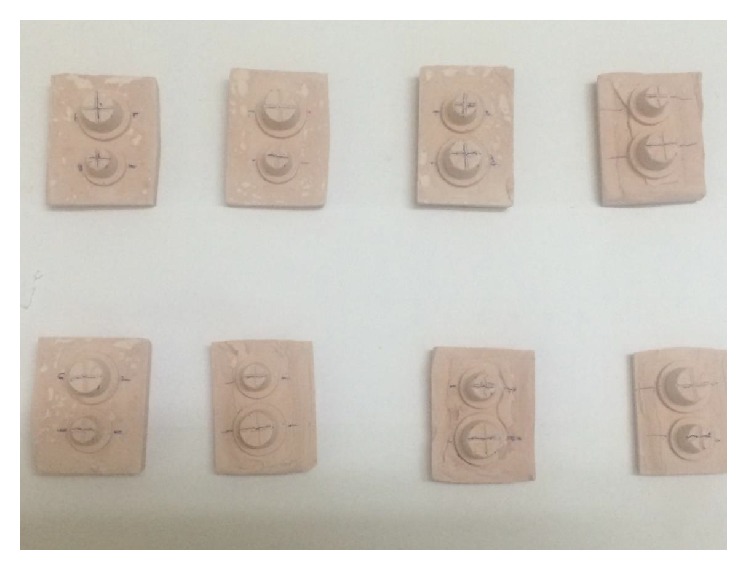
Stone dies obtained from impression.

After the disinfection, the impressions were stored at 25 ± 10°C for one hour for elastic recovery and hydrogen release of vinyl polysiloxane. According to the manufacturer's instruction, 30 gm of preweighed Type IV gypsum was mixed with six mL of distilled water to obtain 0.20 water-powder ratio. The vacuum mixer was utilized for gypsum mixing, and it was poured into the impression under mechanical vibration (Figure 4). The stainless steel retentive struts were attached to the cast base for easy removal of the set cast. The set gypsum cast was removed from the cast after one hour with due care, to avoid the impression damage.

The second, third, and fourth dental casts were obtained following the same procedure after 8- hour, 24-hour, and 3-day interval from the same impression. All the measurements were performed after complete drying of gypsum cast. The measurement of set gypsum cast for linear dimensional change to the reference points was accomplished by traveling microscope with 0.01 *μ*m accuracy. The obtained data obtained was subjected to Analysis of Variance test at significance level <0.05. The percentage of variation of stone dies from the master cast was calculated by the following formula:

(1) Percentage of deviation=mean stone dimension−mean master dimensionmean master dimension×100.

## 3. Results

The present study evaluated the accurate reproducibility of dental die from repeated pour of an impression at the different time intervals.  Table 1 lists the mean values, standard deviation, and percent of deviation of the stone die obtained from two-step putty reline impression technique. The dies obtained from this technique were shorter in height and wider in diameter. There was a progressive reduction of the percentage of variation in the height on repeated pour from −0.36 to −0.97%. The interabutment distance was increased up to 0.40% after repeated pour. The upper diameter of the die was increased by 0.4–0.90%; the lower diameter showed an increased diameter by 0.04–0.12%. The one-way ANOVA showed the significant variation among all the measurements except the upper diameter of abutment with *P* value of 0.424.

Table 2 shows the mean measurement values with the percentage of deviation for die in a heavy-light body one-step impression method. The resultant dies were also shorter and wider in diameter. The abutment height was reduced to the range of −0.73 to −1.21%, while the upper diameter of the abutment was increased by 0.50–0.80%. The interabutment distance between the abutments was having 0.53% variation after pour at 24 hours. The percentage of variation for dies was progressively increased over the repeated pouring of the same impression. *P* value was statistically significant for all measurements except upper diameter of abutment with a value of 0.647.

Table 3 displays the stone die measurement obtained from monophase impression along with the percentage of variation from the master die. The percentage of variation was large in this group in comparison to other methods. The mean percentage of variation for interabutment width was at 1.47%, and the abutment height variation was up to −1.34%. The abutment at upper diameter variation was at 1.20%; lower location was 0.83%. The dimensional accuracy of dies was significantly varied over the repeated pour at abutment upper and lower diameter with *P* value of 0.00 and 0.002, respectively.

Table 4 shows the mean measurement values and percentage of variation for the polyether impression group. This group showed the highest variation in dimension after repeated pour. The resultant dies after repeated pour were shrunk both in diameter and in height. The change for dies varied from −0.36 to −1.46 in height and −0.50 to −1.30 for upper diameter. The interabutment distance was also decreased over the range of −0.46 to −1.13%.

## 4. Discussion

The accurate impression is critical for the fabrication of precise indirect cast restorations. The favorable prognosis of cast restorations is mainly dependent on the well-adapted margins [3]. The multiple pours of the dental impression are required in various situations ranging from inadvertent damage to verification of contact and emergence profile on intact cast [10]. The impression materials and methods that are capable of providing dimensionally accurate die on repeated pour are great advantage to clinicians and laboratory technician. The study explored the commonly used impression methods in their ability to produce the accurate die after the multiple pours. The accuracy of impression material is attributed to multiple factors, including elastic recovery, the direction of setting contraction, continuous polymerization, and evaporation of volatile contents.

The additional silicone impression materials are most commonly used in dentistry due to their better accuracy [11], easy manipulation, and patient acceptance. According to the researchers of the opinion, the impression methods significantly influence the accuracy compared to the material used during the impression. The results from the study reinforced the opinion of the previous researchers; the accuracy of dies varied significantly between different impression methods [12]. Chee and Donovan [13], on a comparison between double mix and single mix putty reline technique, concluded that the double mix two-stage impression provided the more accurate cast. The main disadvantage of single mix technique reported by the researchers was the failure to record the finer details. A few researchers like Hung et al. [14] and Idris et al. [15] reported the insignificant difference between the double mix and single mix technique in accuracy. The study evaluated the two-step putty reline with light body additional silicone impression method. According to the recommendation of earlier researchers, the wash bulk of two mm was utilized during the study for improved dimensional accuracy of dies [16].

The study results showed that the dies obtained of one-step heavy-light body impression and monophase impression were relatively wider in diameter than the master dies in comparison with two-stage putty reline technique. The wider dies on multiple pours were due to the polymerization shrinkage towards the wall of the impression tray. The polymerization shrinkage was least in putty relined technique due to the low matrix and filler ratio [17]. Few researchers recommend the double mix single-step impression to eliminate the inaccuracies in the cast due to deformation and elastic recoil of first-phase material [18]. The reseating and removal for the light body relining process lead to the deformation of already set high viscosity putty material. Hence, the resultant dies from putty reline technique showed a slight variation in dimension. The results from the study also indicate the extended time allowed the recovery of an impression from the strain and improved the accuracy of the dies. The results of the study indicated that the single-step heavy-light body impression technique showed the percentage of variation in the width ranging from 0.04 to 0.90. The subsequent dies obtained from this impression method after multiple pours were significantly inaccurate. The second and the third pour exhibited the percentage of variation of 0.05% and 0.06%, respectively. The dimensional change was slightly higher than putty reline impression technique. The compromised elastic recovery of heavy body impression material is due to the less proportion of filler particle in comparison with the putty material [19]. The inability in maintaining the uniform thickness of light body material also leads to larger dimensional changes [20]. The dies obtained from multiple pours had −0.36 to −0.97% percentage of variation. The results of the study are in agreement with Stackhouse [20]. The shorter dies due to contraction of vertical dimension towards the occlusal preparation with the impression attached to the tray.

The larger section of clinicians prefers the monophase impression material due to its quick and easy impression procedure. The procedure is accomplished in one step utilizing single consistency impression material. The results from the study indicate that the upper diameter of the dies had increased the percentage of variation within the range of 0.70–1.20. The die produced after repeated impression pour was shorter than the master cast by −1.34%. The percentage of variations were significantly higher in comparison to putty reline and single-step heavy-light body impression technique. The results are in compliance with the observation of Millar et al. [21]; they reported the poor dimensional accuracy and high surface defects in monophase impression material because of its relatively high viscosity and reduced flow. Johnson and Craig [8] reported the larger die diameter due to the contraction of impression material towards the walls of the impression tray.

The polyether impression materials continue to be preferred by the clinicians due to its hydrophilic nature, improved flow, and easy manipulation. The rigidity of the polyether is its main disadvantage, leading to difficulty in removing the impression. The dies prepared with polyether impression material were small both in diameter and in height. The dimensional accuracy of the dies on multiple pours was significantly poor. The results could be due to the inability of polyether impression material in its elastic recovery [22]. It is known for good short-term dimensional stability. The water imbibition from gypsum during setting reaction also leads to the dimensional inaccuracy of dies [23].

## 5. Conclusions

Within the limitation of the study, following conclusions were drawn. The putty reline technique (two steps) and heavy-light body additional silicone (1 step) resulted in the lowest percentage of variation from a master model. Monophase impression technique produced the least dimensional accuracy. The shrinkage of impression material leads to the larger diameter and shorter dies. The polyether impression showed the highest distortion over the repeated pour, due to lesser ability to recover. All the impression techniques showed the statistically significant changes in dimension over the repeated impression pour.

## Figures and Tables

**Table 1:** The mean measurement values of stone dies, standard errors, and percentage of errors from master cast from putty reline impression technique.

Measurement	First pour (1 Hr)			Second pour (12 Hr)			Third pour (24 Hr)			Fourth pour (48 Hr)			*P*
	Mean	SD	%	Mean	SD	%	Mean	SD	%	Mean	SD	%	
Upper dia-abutment1	10.03	0.13	0.40	10.04	0.05	0.50	10.08	0.02	0.90	10.06	0.01	0.70	0.424
Lower dia-abutment1	15.65	0.03	0.12	15.65	0.04	0.12	15.71	0.05	0.05	15.66	0.05	0.04	0.000
Interabutment distance	14.96	0.03	0.20	14.98	0.14	0.27	15.02	0.18	0.0	14.99	0.05	0.40	0.000
Height abutment1	8.17	0.04	−0.36	8.15	0.03	−0.60	8.12	0.03	−0.97	8.16	0.03	−0.48	0.000

**Table 2:** The mean measurement values of stone dies, standard errors, and percentage of errors from master cast from heavy-light body single mix impression technique.

Measurement	First pour (1 Hr)			Second pour (12 Hr)			Third pour (24 Hr)			Fourth pour (48 Hr)			*P*
	Mean	SD	%	Mean	SD	%	Mean	SD	%	Mean	SD	%	
Upper dia-abutment1	10.04	0.04	0.50	10.05	0.05	0.60	10.08	0.06	0.90	10.07	0.03	0.80	0.647
Lower dia-abutment1	15.66	0.03	0.19	15.65	0.14	0.12	15.74	0.16	0.03	15.74	0.13	0.02	0.000
Interabutment distance	14.98	0.04	0.04	15.01	0.04	0.20	15.04	0.05	0.53	14.99	0.03	0.40	0.008
Height abutment1	8.14	0.04	−0.73	8.14	0.06	−0.73	8.11	0.05	−1.09	8.10	0.05	−1.21	0.020

**Table 3:** The mean measurement values of stone dies, standard errors, and percentage of errors from master cast from Monophase impression technique.

Measurement	First pour (1 Hr)			Second pour (12 Hr)			Third pour (24 Hr)			Fourth pour (48 Hr)			*P*
	Mean	SD	%	Mean	SD	%	Mean	SD	%	Mean	SD	%	
Upper dia-abutment1	10.06	0.08	0.70	10.09	0.07	1.00	10.13	0.07	1.20	10.10	0.05	−0.70	0.000
Lower dia-abutment1	15.71	0.08	0.51	15.76	0.07	0.63	15.80	0.07	0.83	15.78	0.07	0.70	0.002
Interabutment distance	15.12	0.05	1.27	15.15	0.06	1.47	15.15	0.05	1.47	15.13	0.03	0.73	0.074
Height abutment1	8.16	0.17	−0.48	8.14	0.07	−0.73	8.09	0.08	−1.34	8.12	0.11	−0.97	0.124

**Table 4:** The mean measurement values of stone dies, standard errors, and percentage of errors from master cast from polyether impression technique.

Measurement	First pour (1 Hr)			Second pour (12 Hr)			Third pour (24 Hr)			Fourth pour (48 Hr)			*P*
	Mean	SD	%	Mean	SD	%	Mean	SD	%	Mean	SD	%	
Upper dia-abutment1	9.94	0.08	−0.50	9.86	0.77	−1.30	9.88	0.06	−1.10	9.89	0.05	−1.00	0.012
Lower dia-abutment1	15.56	0.08	−0.44	15.53	0.07	−0.63	15.52	0.07	−0.70	15.55	0.05	−0.51	0.031
Interabutment distance	14.86	0.10	−0.46	14.79	0.21	−0.93	14.76	0.03	−1.13	14.82	0.03	−0.73	0.181
Height abutment1	8.17	0.20	−0.36	8.08	0.18	−1.46	8.11	0.15	−1.09	8.15	0.10	−0.60	0.003

## References

[1] Brunton P. A., Sharif M. O., Creanor S., Burke F. J. T., Wilson N. H. F. (2012). Contemporary dental practice in the UK in 2008: indirect restorations and fixed prosthodontics. *British Dental Journal*.

[2] Devan M. M. (2005). Basic principles in impression making. *Journal of Prosthetic Dentistry*.

[3] Larson T. D. (2012). The clinical significance of marginal fit. *Northwest Dentistry*.

[4] Donovan T. E., Chee W. W. L. (2004). A review of contemporary impression materials and techniques. *Dental Clinics of North America*.

[5] Chugh A., Arora A., Singh V. P., Das U. M., Marwah N., Toumba K. (2012). Accuracy of different putty-wash impression techniques with various spacer thickness. *International Journal of Clinical Pediatric Dentistry*.

[6] Nissan J., Gross M., Shifman A., Assif D. (2002). Effect of wash bulk on the accuracy of polyvinyl siloxane putty-wash impressions. *Journal of Oral Rehabilitation*.

[7] Luthardt R. G., Walter M. H., Quaas S., Koch R., Rudolph H. (2010). Comparison of the three-dimensional correctness of impression techniques: a randomized controlled trial. *Quintessence International*.

[8] Johnson G. H., Craig R. G. (1985). Accuracy of four types of rubber impression materials compared with time of pour and a repeat pour of models. *The Journal of Prosthetic Dentistry*.

[9] Kumar V., Aeran H. (2012). Evaluation of effect of tray space on the accuracy of condensation silicone, addition silicone and polyether impression materials: an in vitro study. *Journal of Indian Prosthodontist Society*.

[10] Patil P. G. (2011). Modified soft tissue cast for fixed partial denture: a technique. *Journal of Advanced Prosthodontics*.

[11] Gonçalves F. S., Popoff D. A., Castro C. D., Silva G. C., Magalhães C. S., Moreira A. N. (2011). Dimensional stability of elastomeric impression materials: a critical review of the literature. *European Journal of Prosthodontics and Restorative Dentistry*.

[12] Caputi S., Varvara G. (2008). Dimensional accuracy of resultant casts made by a monophase, one-step and two-step, and a novel two-step putty/light-body impression technique: an in vitro study. *Journal of Prosthetic Dentistry*.

[13] Chee W. W. L., Donovan T. E. (1992). Polyvinyl siloxane impression materials: a review of properties and techniques. *The Journal of Prosthetic Dentistry*.

[14] Hung S. H., Purk J. H., Tira D. E., Eick J. D. (1992). Accuracy of one-step versus two-step putty wash addition silicone impression technique. *The Journal of Prosthetic Dentistry*.

[15] Idris B., Houston F., Claffey N. (1995). Comparison of the dimensional accuracy of one- and two-step techniques with the use of putty/wash addition silicone impression materials. *The Journal of Prosthetic Dentistry*.

[16] Nissan J., Laufer B.-Z., Brosh T., Assif D. (2000). Accuracy of three polyvinyl siloxane putty-wash impression techniques. *Journal of Prosthetic Dentistry*.

[17] Chen S. Y., Liang W. M., Chen F. N. (2004). Factors affecting the accuracy of elastometric impression materials. *Journal of Dentistry*.

[18] Chee W. W., Donovan T. E. (1989). Fine detail reproduction of very high viscosity poly (vinyl siloxane) impression materials. *The International Journal of Prosthodontics*.

[19] Carlo H. L., Fonseca R. B., Soares C. J., Correr A. B., Correr-Sobrinho L., Sinhoreti M. A. C. (2010). Inorganic particle analysis of dental impression elastomers. *Brazilian Dental Journal*.

[20] de Araujo P. A., Jorgensen K. D. (1985). Effect of material bulk and undercuts on the accuracy of impression materials. *The Journal of Prosthetic Dentistry*.

[21] Millar B. J., Dunne S. M., Robinson P. B. (1998). In vitro study of the number of surface defects in monophase and two-phase addition silicone impressions. *The Journal of Prosthetic Dentistry*.

[22] Endo T., Finger W. J. (2006). Dimensional accuracy of a new polyether impression material. *Quintessence International*.

[23] Kanehira M., Finger W. J., Endo T. (2006). Volatilization of components from and water absorption of polyether impressions. *Journal of Dentistry*.

